# Exploring community awareness and perspectives on zoonotic disease in Nepal: a qualitative study

**DOI:** 10.1186/s12889-026-28672-8

**Published:** 2026-08-11

**Authors:** Anna Durrance-Bagale, Hari Basnet, Nanda Bahadur Singh, Steven R Belmain, James W Rudge, Natasha Howard

**Affiliations:** 1 Department of Global Health & Development, London School of Hygiene & Tropical Medicine, 15-17 Tavistock Place, London, WC1H 9SH UK; 2 Nepalese Ornithological Union, Kathmandu, Nepal; 3 Central Department of Zoology, Tribhuvan University, Kathmandu, Nepal; 4 Natural Resources Institute, University of Greenwich, Chatham Maritime, Kent, ME4 4TB UK; 5 Nuffield Centre for International Health & Development, Leeds Institute of Health Sciences, University of Leeds, LS2 9NL Leeds, United Kingdom; 6 Saw Swee Hock School of Public Health, National University of Singapore, National University Health System, 12 Science Drive 2, Singapore, Singapore

**Keywords:** Awareness, Community, Infectious disease, Nepal, Risk factors, Zoonotic disease

## Abstract

**Background:**

Qualitative research focused on Nepalese community perspectives on zoonotic disease is lacking. We aimed to characterise the contextual factors and mechanisms driving awareness of risk and prevention of zoonotic diseases in selected communities in Nepal.

**Methods:**

We conducted 39 semi-structured individual interviews in six communities, and five focus groups with 34 participants. Ten participated in photovoice. Twenty healthcare professionals and policymakers were interviewed, 14 representing human health and six representing animal health. We analysed data thematically.

**Findings:**

We generated two major themes: disease awareness, and beliefs and behaviours. Participants were aware of diseases that they had directly experienced or that they perceived might affect their family or livelihood. This was especially true of rabies. Information on disease was usually spread informally. Use of traditional medicine was widespread: some participants saw this behaviour as anachronistic, with others seeing it as pragmatic, as accessing traditional healers is often easier than visiting health posts. Consuming bushmeat was seen as something ‘others’ did, although some suggested bushmeat was medicinal and others distinguished between ‘clean’ and ‘dirty’ rodents. Hygiene practices were perceived as necessary to remove dirt but seldom linked explicitly to illness. Religious beliefs were presented as a reason for not harming rodents.

**Conclusion:**

Working with communities is essential to understanding complex behaviours that might increase disease risk, especially in communities that lack resources to mitigate these threats. Specific implications for policymakers are the importance of ensuring the healthcare system is inclusive of different groups needing its services, and that differing beliefs are considered when designing services.

**Supplementary Information:**

The online version contains supplementary material available at https://doi.org/10.1186/s12889-026-28672-8.

## Introduction

The COVID-19 pandemic highlighted the potential for diseases of zoonotic origin to cause serious health and economic issues and threaten livelihoods globally [1, 2]. An estimated 75% of new or emerging infectious diseases affecting humans are of zoonotic origin, with many of these emerging from wildlife reservoirs [1, 3, 4], highlighting the threat that these pathogens pose globally [5]. Anthropogenic drivers of zoonotic disease emergence include rapid population growth, urbanisation, habitat encroachment, fragmented landscapes, proximity of humans to animals [6, 7], and bushmeat consumption [8, 9], further complicated by under-resourced and inaccessible healthcare provision [3, 6, 10–13]. Examining the human-animal interfaces that facilitate this emergence and potential cross-species transmission is essential to preventing spread of zoonotic disease [2, 14, 15].

Zoonotic disease outbreak risk in Nepal is affected by environmental, socio-economic and other anthropogenic factors [3]. Nepal is categorised as a lower-middle income economy by the World Bank, with a gross national income per capita of US$1,086 − 4,255 [16]. In 2019, Nepal spent 4.45% of gross domestic product on healthcare, averaging US$53 per capita [17]. Many people in rural and urban areas of Nepal are subsistence or backyard farmers, raising livestock and poultry, increasing the risk of disease transmission. This is particularly relevant in rural and agricultural areas, where interactions between wildlife reservoirs and domesticated livestock can create opportunities for pathogen transmission [18, 19].

Healthcare services in Nepal are basic, and a large portion of the population lacks access to qualified healthcare providers, especially in remote and rural areas. For example, in 2011 it was estimated that 41% of rural communities in the Terai region of the country have no access to a health post, and 80% cannot reach a public hospital within 30 min by public transport [20]. This lack of access to healthcare provision means that communities are unlikely to report potential spillover events, and this, coupled with lack of government-approved prevention measures and poorly functioning or non-existent surveillance mechanisms means that the presence of disease may be unrecorded [18, 21]. In terms of access to veterinary services, in 2019 it was estimated that only 3–5% of livestock owners in Nepal had contact with a qualified veterinarian, and most of those were people living in urban areas [22].

Our scoping review, published in 2021 and focused on the Indian subcontinent, found that the most frequently discussed potential driver of zoonotic disease risk was lack of public awareness [15]. Official information through healthcare workers or other channels was often unavailable, and people instead relied on informal networks of neighbours, relatives and friends. However, access to information and awareness of routes of disease transmission was not reflected in behaviours [15, 19]. For example, although rabies is recognised as a fatal disease by many people in the subcontinent, they reported an unwillingness to access post-exposure prophylaxis following contact with an animal, even when treatment is free (as it is in Nepal) [15]. This suggests that complex, non-explicit processes may underlie behaviours that appear, on the surface, to be irrational [23]. By discussing relevant issues around zoonotic disease, awareness and behaviours with community participants, we sought to build a picture of what people understand (observable) and how this relates to how they do, or do not, behave (the ‘real’ world). Working with communities, exploring behaviours and drivers that may facilitate transmission in context with practices and cultural beliefs is crucial to comprehending and decreasing risk of new or re-emerging zoonotic disease spread [24, 25].

Studies have examined awareness and practices around zoonotic disease in smallholder farmers in Nepal [19, 26, 27], but research on zoonotic disease in general is lacking in Nepal, particularly work that does not attempt to quantify risk but instead focuses on elucidating what community members know about these diseases. As zoonotic risk occurs first at community level, it is essential that understanding of local perspectives on these diseases and relevant barriers or enablers to potential mitigatory responses to these is developed. Considering these knowledge gaps, this study aimed to characterise the key contextual factors and mechanisms driving awareness of risk and prevention strategies for zoonotic diseases in selected communities in Nepal.

## Methods

### Study design

We conducted a qualitative case study of zoonotic disease perspectives in Nepali communities, drawing from interviews and focus group discussions (FGDs) with members of communities, health-workers, veterinarians, and policymakers, and photovoice with community members. This design enabled in-depth exploration of complex phenomena within the specific community context, including “how” and “why” of perspectives, allowing us to gather and analyse rich data from multiple sources, such as interviews and observations, to build a more holistic understanding of lived experiences and perceptions than numerical data alone can provide. This study was part of ADB’s PhD research, which focused on drivers of zoonotic spillover in Nepal.

### Study sites

**Fig. 1:**
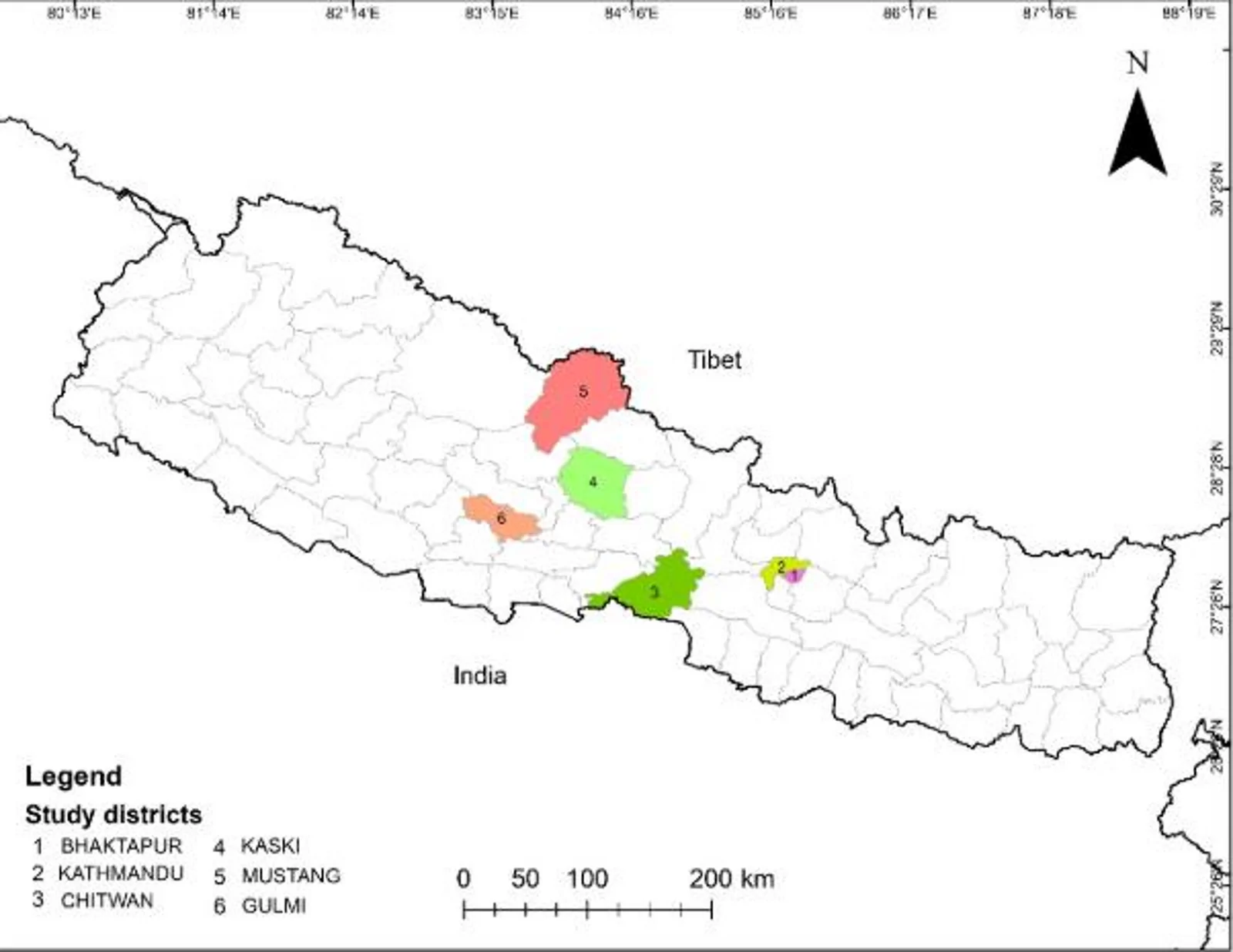
Map of study sites

We purposively selected six sites (two in the Kathmandu valley; Fig. 1) after discussion with Nepali colleagues. These sites were chosen as they represent urban and rural areas and different regions of Nepal, with potential differences in experience between castes and ethnicities, and were all likely to enable us to identify participants who could provide us with rich information on the topics in which we were interested. Bhaktapur (site 1) is a densely populated city 13 km east of central Kathmandu. Around 90% of the population is Newar, a distinct group with its own language, Newari, and this was reflected by the fact that most of the Bhaktapur participants identified themselves as Newar. An informal settlement in central Kathmandu along the banks of the Bagmati river was selected as site 2. This settlement contained approximately 130 households, with a few small shops. Most participants were unemployed. Site 3 (Chitwan) is situated in a national park area in the lowland Terai region, bordering India, where many people earn a living from tourism centred on elephant riding and birding. Many of the participants at this site were involved in the tourism industry. Site 4 (Pokhara) is the second-largest city in Nepal, with a well-developed tourist infrastructure. Participants were living in a smaller settlement on the outskirts of the city, often farming small landholdings, but with easy access to the city. Site 5 (Mustang) is a remote, mountainous, sparsely populated area. Participants lived in one of the regional villages, with a population of around 1500. Most were small-scale farmers or ran small guesthouses or restaurants catering to domestic tourists. Site 6 (Gulmi) is a relatively remote, rural, mountainous area where most people are small-scale farmers. Participants stated that roads were being built and infrastructure was developing rapidly in their area. In terms of religion, most study participants stated that they were Hindu, while others identified themselves as Buddhist, particularly in Mustang. All participants spoke fluent Nepali.

### Participant sampling and recruitment

We used a combination of typical sampling (a type of purposive sampling, where participants who may generate informative or interesting data are selected) and snowballing, where participants recommend other potential participants. As with most sampling used in qualitative research, typical sampling does not allow us to generalise findings, but instead to identify usual experiences in a particular setting the implications of which may be transferred or more broadly relevant [28, 29]. To be eligible, potential participants had to be aged at least 18 and give informed consent. We enrolled adult participants and aimed to have six to eight individual interviews and run one FGD with six to 10 participants per site. This initial estimated sample size was judged sufficient to gain an understanding of the views, opinions and experiences of participants, but was open to adjustment during the study if this became necessary (e.g., if we were not collecting informative data) [28]. In each district selected, we contacted a healthcare worker (if available) and asked them to suggest people to contact in each community. If we could not contact a healthcare worker, we instead attempted to identify a prominent member of the community, for example a teacher or member of a community group (e.g., a local women’s group) and asked them to suggest participants. For the six sites, the initial contacts were a male teacher (Mustang), female leader of a local community group (Bhaktapur), member of the local women’s group (Gulmi, Chitwan), male veterinarian (Kaski), and male doctor (Kathmandu informal settlement). ADB and HB met the suggested people, explained the aims of the study, and interviewed them if they were willing to participate. We asked each participant to identify another potential participant (snowballing) until approximately six to eight individuals and one group had agreed to participate and had been recruited per community. We determined data saturation and stopped collecting data once we judged that no new topics or information were being discussed in interviews and FGDs. We discussed with potential participants that their participation was entirely voluntary, and checked to ensure that they understood that if they did not want to agree to participate this would not disadvantage them in any way. Before beginning the interview we also made it clear that they could stop participating at any point, and were free to do so without any penalty or disadvantage. We also stated that if they had begun an interview and then decided not to continue, we would delete the audio-recording of the interview.

ADB generated a seed list of Nepali human or animal healthcare professionals and national or regional policymakers, and asked colleagues in Nepal to nominate potential interviewees from appropriate organisations in the country. All were contacted by email, with two refusing due to time constraints and three not responding. At the end of their interview participants were asked to nominate others who might have useful experiences or viewpoints to discuss.

### Consent procedures

ADB explained the aims of the study to potential participants, including the fact that it was part of her PhD research, and why we were interested in this particular topic, before any other study procedure took place. If they agreed to participate, they were asked to sign a consent form or give verbal consent confirming that they had read and understood the information sheet. Consent of illiterate participants was witnessed by a person unrelated to the study team and selected by the participant, after thorough explanation of the relevant documents. A separate consent form was provided for people who appeared in any photographs taken by participants. If consent was not provided, the image was to be immediately deleted. In practice, no participant photographed a person during the study.

### Data collection

Data were collected between April and July 2022. Both interviewers (ADB [PhD] and HB [MSc]) had post-graduate training in social science research and interview methods and were working as researchers. All participants were interviewed once in their home, place of work or a local café, as they judged appropriate, and to improve confidentiality and privacy. Only the participant and the research team were present during the interview. All sessions were recorded with the permission of the participant(s).

### Interviews

Semi-structured interviews are frequently used in qualitative research as they allow researcher and participant some flexibility during the interview and also allow focus on issues that either consider are important and relevant to the research [30, 31]. This type of interviewing has been defined as designed to encourage ‘descriptions of the life world of the interviewee in order to interpret the meaning of the described phenomena’ [31].

Topic guide development was informed by findings from a literature review we previously published [15] and our contextual understanding. We revised topic guides iteratively during the interviewing stage as needed. Colleagues in Nepal reviewed topic guides for relevance and socio-cultural appropriateness before interviews began. The topic guide for community participants covered human-animal contact, biosecurity and food hygiene, environmental changes, health issues, and disease awareness, including any experience with awareness programmes. The topic guide for policymakers and practitioners covered participants’ relevant experience, views on community awareness, and comprehension of governmental policy on zoonotic and infectious diseases. As we were interested in the opinions of participants, rather than having a list of specific questions that we asked one-by-one the topic guides were broad and allowed discussion of additional relevant topics raised by participants, as appropriate. The topic guide is available from the first author on request.

Interviews lasted around 30 min and were conducted in English (by ADB) or Nepali (by HB and ADB) as per participant preference. Seven of the healthcare professional and policymaker interviews were conducted remotely by ADB using Zoom software (Zoom Video Communications Inc, San Jose) and audio-recorded with automatic transcription enabled. ADB reviewed and corrected the transcripts against the recording.

### FGDs

Focus group discussion can be more dynamic than semi-structured, individual interviews [30, 31]. They allow participants to have a more organic discussion and share perspectives, with the researcher prompting as necessary but otherwise allowing discussion to flow. Concepts not included in the topic guide can be raised and discussed by participants, allowing generation of both individual and collective ideas. The topic guide for the FGDs covered the same topics as that for the individual interviews. Discussions lasted around 45–60 min and were conducted in Nepali. One FGD was conducted in a mixture of Nepali and Newari at participants’ request as all participants were fluent in both languages.

### Unstructured observations

During the interviews and FGDs ADB noted brief observations on the ‘three Cs’ [32]: any context, content or concept that appeared potentially significant or interesting, e.g., how participants interacted with animals around them, or if they laughed or appeared uncomfortable while talking about certain topics. This type of unstructured observation can provide insight into contextual factors and how these may relate to topics being discussed, or actions being taken [32].

### Photovoice

At the end of their interview, participants who had engaged well and were enthusiastic about participating were asked to use a simple digital camera to take photographs that illustrated their perceptions, feelings and beliefs around zoonotic disease risk in their community. They were then asked to describe why they had taken these images.

Photovoice is a participatory research method that can be used as a collaborative approach to data collection [33]. Participants take photographs and discuss the significance of these images for them in their particular context and situation [34]. This method can enhance understanding of community experiences, beliefs, behaviours and priorities [35] and may be particularly useful in marginalised communities who are likely less familiar with traditional methods of enquiry [36]. Photovoice physically involves participants rather than them being simply subjects of the research. This type of collaboration is key to allowing participants to potentially demonstrate their beliefs, practices, and traditions, and their multiple roles, for example as a community member, mother, member of women’s group, farmer, business owner, and disposer of dead animals. Participants are able to demonstrate how they make sense of their world: what is (non-)significant for them and what they would like to know to allow them to have agency over their situation and the world around them [36].

### Data management and analysis

We considered data quality in terms of credibility, confirmability, and transferability. For credibility (i.e., believability and trustworthiness), we followed May and Pope’s (2000) approach, often used in healthcare research, of reflexivity, triangulation, participant validation, transparency, deviant voices, and relevance [37]. For confirmability (i.e., appropriate methodology), we followed Silverman’s framework of reliability checking, audit trail, comprehensive data use, seeking minority perspectives, and extraction from original sources [38]. We did not use double-coding, as this study was conducted as part of a PhD thesis and instead relied on reliability checks and discussion with supervisors. For transferability (i.e., broader meaning), we focused on naturalistic or conceptual extendibility of our findings to other settings.

All recordings and photographs were given an alphanumeric code to ensure confidentiality. Completed consent forms were scanned and shredded. Electronic data were password-protected and only accessible to the study team.

Recordings in Nepali were transcribed into English by a Nepali healthcare professional familiar with public health and infectious disease, and who was fluent in both languages. Two of the first transcriptions were reviewed and back-translated by a second native Nepali speaker to ensure that the transcription accurately reflected the recording. Interviews in English were transcribed by ADB. Names used by participants for animal diseases were cross-checked with a Nepali veterinarian fluent in English to ensure that they represented the correct disease as far as possible, and to check for any nuance related to these diseases that might not have been obvious to the translator [39]. Transcripts were not returned to participants.

Interview and FGD transcripts were imported into NVivo software (QSR International Pty Ltd, Version 12, 2018) for data management. Photographs were given an identifying code and simple textual description by ADB. This description was imported into NVivo with the accompanying explanatory text from the participant who took the photograph. Unstructured observation notes were imported into NVivo and used to generate a thicker, richer description of the phenomena being discussed. Thick description uses data from different sources to contextualise events and behaviour and aid in their interpretation [40].

ADB reviewed and coded all transcripts, explanations of photographs, and observation notes in NVivo, using inductive reflexive thematic analysis to generate themes and sub-themes from the data. The analysis involved six steps: (i) data familiarisation; (ii) generating initial codes; (iii) generating themes; (iv) reviewing potential themes; (v) defining/naming themes; and (vi) synthesising findings [41, 42].

### Reflexivity

These interviews were often the first time many participants were asked about their views and experiences on similar issues by anyone, particularly an obvious outsider. This outsider status can have advantages, as ‘insiders’ (i.e., people who are part of the community involved in the research) may be too close and their personal experience may affect their expectations or the way they interact with participants [43]. It may also have disadvantages, such as participants experiencing shyness or reticence to discuss what they might perceive as ‘sensitive’ topics with someone from outside their culture. We discuss this further in ‘Implications’ and ‘Study strengths and limitations’ sections.

Producing research and knowledge, interpreting findings and writing up these findings into a coherent form, is never neutral or objective but always includes values, judgements and assumptions, whether implicit or explicit [44, 45]. Researchers must consider the potential impact of their beliefs, biases, experiences and emotions during the life of the research project [46], particularly if they are writing up their findings and conclusions from the perspective of their own, ‘foreign gaze’ [47]. ADB, a white, English woman, and HB, a Nepali man, who conducted the fieldwork together, have worked together on previous projects and have a good rapport. At the end of each research day we discussed our findings and any issues that had arisen. Participants appeared to be comfortable discussing topics with us, and they were reminded that if there was anything they did not feel comfortable discussing then they could end the interview at any time. ADB kept her positionality (cultural assumptions, preconceptions and values) in mind throughout the fieldwork and data analysis in an attempt to mitigate the effects of these. HB is from a rural area of the west of Nepal and has spent many years living in Kathmandu. He was often a different caste and background to the participants but was effective at making participants feel comfortable and encouraging them to talk about their experiences.

### Ethics

The Nepal Health Research Council (ref: 2193, approval granted 23 February 2022) and London School of Hygiene & Tropical Medicine Observational Research Ethics Committee (ref: 26507, approval granted 16 March 2022) provided ethics approval. All research was conducted in accordance with national ethical and research guidelines and with relevant permissions. In publications from this study, participants were only identified by location to help preserve confidentiality.

## Findings

### Participant characteristics

Thirty-nine people (21 men, 18 women) from communities at six sites participated in semi-structured interviews. Thirty-four people (14 men, 20 women) participated in five FGDs at different sites (Table 1). Two people approached refused to participate, due to lack of time. Estimated ages are given as some participants were unable or unwilling to disclose their age. Photographs were taken by nine interviewees and one FGD participant across the six sites. This was judged to be a sufficient number to demonstrate the utility of photovoice as a method in this exploratory study, and does not imply saturation. Twenty Nepali healthcare professionals and policymakers were interviewed in English (Table 2): 14 representing human health (13 men, one woman) and six representing animal health (three men, three women). Location information is not included for these 20 participants to protect their anonymity.

**Table 1:** Community participant characteristics

Identifier	Gender	Estimated age	Language
Bhaktapur1	Female	45–50	Nepali
Bhaktapur2	Female	50–55	Nepali
Bhaktapur3	Male	35–40	Nepali
Bhaktapur4	Female	30–35	English
Chitwan1	Male	45–50	Nepali
Chitwan2	Male	70–75	Nepali
Chitwan3	Female	45–50	Nepali
Chitwan4	Male	40–45	English
Chitwan5	Male	35–40	Nepali
Chitwan6	Male	60–65	Nepali
Gulmi1	Female	65–70	Nepali
Gulmi2	Female	35–40	Nepali
Gulmi3	Female	55–60	Nepali
Gulmi4	Female	45–50	Nepali
Gulmi5	Male	60–65	Nepali
Gulmi6	Female	55–60	Nepali
Kathmandu1	Male	25–30	Nepali
Kathmandu2	Male	45–50	Nepali/English
Kathmandu3	Female	35–40	Nepali
Kathmandu4	Female	35–40	Nepali
Kathmandu5	Male	20–25	English
Mustang1	Female	45–50	Nepali
Mustang2	Female	40–45	Nepali
Mustang3	Male	20–25	Nepali
Mustang4	Male	40–45	Nepali
Mustang5	Female	35–40	Nepali
Mustang6	Male	40–45	Nepali
Mustang7	Male	45–50	Nepali
Pokhara1	Male	45–50	Nepali
Pokhara2	Male	20–25	Nepali
Pokhara3	Male	30–35	Nepali
Pokhara4	Female	50–55	Nepali
Pokhara5	Male	45–50	Nepali
Pokhara6	Female	50–55	Nepali
Pokhara7	Male	55–60	Nepali
Pokhara8	Male	55–60	Nepali
Pokhara9	Female	50–55	Nepali
Pokhara10	Female	35–40	Nepali
Pokhara11	Male	55–60	Nepali
Bhaktapur FGD	5 male, 4 female	20–70	Nepali/Newari
Chitwan FGD	1 male, 3 female	20–70	Nepali
Gulmi FGD	0 male, 9 female	20–70	Nepali
Mustang FGD	5 male, 1 female	20–70	Nepali
Pokhara FGD	3 male, 3 female	20–70	Nepali

**Table 2:** Healthcare professional participant characteristics

Identifier	Type	Gender	Interview
Health1	Infectious disease specialist	Male	In-person
Health2	Clinician/NGO	Male	In-person
Health3	Public health specialist	Male	In-person
Health4	Consultant for health NGOs/iNGOs	Male	In-person
Health5	Government/NGO	Female	In-person
Health6	Infectious disease specialist	Male	In-person
Health7	Government/clinician	Male	In-person
Health8	Consultant for health NGOs/iNGOs	Male	In-person
Health9	Government/infectious disease specialist	Male	In-person
Health10	Consultant for health NGOs/iNGOs	Male	Remote
Health11	Infectious disease specialist/academic	Male	Remote
Health12	Public health specialist	Male	Remote
Health13	Consultant for health NGOs/iNGOs	Male	Remote
Health14	Government/public health specialist	Male	Remote
Livestock1	Government/veterinarian	Male	In-person
Livestock2	Government/veterinarian	Male	In-person
Livestock3	Government/veterinarian	Female	In-person
Livestock4	Government/veterinarian	Male	In-person
Livestock5	Government/veterinarian	Female	Remote
Livestock6	Government/veterinarian	Female	Remote

*iNGO* International non-governmental organisation, *NGO* Non-governmental organisation

### Thematic findings

We generated two overarching themes from transcripts and photographs: (i) disease awareness, which included four sub-themes, and (ii) beliefs and behaviours, which also included four sub-themes. In general, reported experiences and opinions were similar between sites, despite potential differences in caste or ethnic group, and no site appeared notably different to any other.

#### Disease awareness

This theme included four sub-themes: experience of disease, perceptions of rabies, livestock and disease, and sources of information. We found that participants were pragmatic: aware of diseases that they perceived might affect their family or livelihood (e.g., rabies, avian influenza) or that they had experienced themselves. This was especially true of rabies, with most participants able to discuss disease transmission, and pre- and post-bite actions. Information on disease was usually spread informally between networks of friends and family, with little experience of formal programming by official organisations.

##### Experience of disease

Disease awareness was related to experience: if participants were aware of zoonoses this was usually a result of caring for domesticated animals (buffalo, goat, poultry) or having contact with dogs or rodents, rather than experience of or with a specific disease:

‘I knew that you don’t have rabies from rodents, but then there could be other diseases related with its faeces. So plague and new things, but we have never experienced those things in our lifetime.’ [Bhaktapur4].

Many participants kept pet dogs or were exposed to community dogs and knew about rabies. Most people knew that rabies was spread by dog bites and fatal, and many understood that vaccination was important. People keeping backyard poultry knew about bird flu:

‘I am aware [*of animal-human transmission*]. The cows, buffalos and other animals have a disease called luto [*demodicidosis*]. If we touch that wound, we might be infected[…]I also was bitten by a dog and I got the vaccine, also the dog was well vaccinated just in case.’ [Chitwan6].

‘I know that dog bites can cause rabies for which rabies vaccine are provided. We are concerned about rabies so we do vaccinate our dog. We sometimes wash our hands after petting them.’ [Mustang3].

One participant who had experienced a dog bite shared knowledge of rabies:

‘The dogs had bitten me and I had taken medicine and vaccine. I still have the bite mark. Dogs can cause rabies and if the infected dogs bite an individual, we need to get vaccinated if the dogs die within 7 days.’ [Gulmi2].

One healthcare practitioner also suggested that awareness was often related to participant experience:

‘If you talk about the communities like if there are some professionals or maybe some workers are engaging with the animals like meat handlers or poultry, poultry farm workers, and poultry farm owners and then maybe the fishermen, and also the mahouts, and some pig farmers, if you go to the communities where this kind of operational activity is going on, maybe you will find some awareness of that because they might be suffering from maybe foot and mouth disease, some skin disease in animals or tuberculosis sometimes, or avian influenza in birds.’ [Health2].

One animal health professional described work he had done with communities experiencing avian influenza, where there was low-level awareness:

‘One of our questions is, are you aware about the diseases that have been transmitted to people and animal? They say yes, sometimes and most of them name like bird flu, but more than that, they don’t know anything.’ [Livestock2].

One practitioner suggested that, although most community participants might not be confident about their knowledge of specific zoonotic diseases, they have a more general understanding:

‘Most of the community don’t know about specific disease. But while talking about the symptoms, do you have these kinds of symptoms? do you have this problem?, they will definitely say, yes we have, and we can identify if it is a communicable disease. But they don’t know the disease by name.’ [Health12].

This was seconded by an animal health representative:

‘Since it’s like after every monsoon, this type of problem, there might be people have some knowledge on that, but definitely as *Leptospira* they don’t have that.’ [Livestock2].

This was reflected by some participants, eager to say they were aware of the possibility of disease transmission from animals, but when probed were unable to be specific:

‘In general I know but being specific like this disease transmit from these animals, I don’t know.’ [Bhaktapur3].

‘I don’t know the name of disease but I just heard that disease can transmit from animals.’ [Gulmi1].

Some participants downplayed the knowledge they did have, even when this clearly informed their behaviours:

‘I don’t have much information but I have seen a man die about 20 years back in Gorkha when a mad dog bites human. I don’t know the name but the dogs who are salivating when they bite can kill humans and they themselves can die as well. Medication has also to be taken fast in order to prevent it[…]We vaccinate the dog so that they don’t spread the disease if they bite other people. People also ask if the dog is vaccinated in case the dog scratches them.’ [Pokhara8].

This was also apparent in an FGD, when a participant appeared to contradict themselves:

‘We have no idea [*about animal-human transmission*]. I had heard that some diseases are transmitted by dogs such as luto [*demodicosis*].’ [Pokhara FGD].

Some participants were happy to acknowledge that, although suggesting they had little awareness, they knew enough to protect themselves and this was reflected in their behaviours:

‘Yes, from rat also disease is transmitted[…]I don’t know [*which one*] but we don’t eat the food that is touched by the rat.’ [Pokhara6].

‘I have no idea about the disease spread by animals but am aware that proper sanitation should be maintained while handling them. I don’t have any idea about that [*rat-borne disease*]. We just clean the rice and other grain properly before eating and ensure that it is stored properly.’ [Pokhara8].

##### Perceptions of rabies

There was general agreement among human and animal healthcare professionals that most people in Nepal are aware of rabies and routes of transmission, but are unwilling, or unable due to lack of time and money, to have rabies prophylaxis, or visit a health post to be vaccinated post-bite. This is despite the fact that rabies vaccination is free in Nepal:

‘I think people in the community level they are more aware about rabies. Because they know dogs are domestic animals, the people in Nepal, they usually have dogs, and they even know about bat. So, I think rabies is the most common zoonotic disease people have heard and also they know about the rabies vaccine, which is available through different public hospitals.’ [Health3].

Demonstrating that the COVID-19 pandemic has increased awareness, one of the informal settlement participants indirectly linked COVID-19 and rabies, stating that he became aware of the potential for zoonotic disease 2 years before the interview, during the pandemic. He displayed understanding of transmission risks, despite having no formal education:

‘I came to know this [*animal-human transmission*] just 2 years ago. And people are staying 2 metres far from each people because disease can even be transmitted from animals. So, there’s no doubt about transmission of disease from different animals like dogs and cats. It’s compulsory to inject rabies vaccine after dog bite. From vaccinated dogs, diseases don’t get transmitted.’ [Kathmandu2].

As community dogs are frequently present in streets throughout the country, people are likely to be exposed to them and may have a better understanding of this disease and behaviours that can prevent the spread (or what actions they should take after a bite). However, this is not always the case. In the informal settlement we visited in Kathmandu, we observed a 2-year-old child cuddling and kissing a community dog, which was drooling and covered in suppurating sores, as the mother sat watching and smiling. This suggests it is unlikely that mother or child had experienced negative effects of such behaviour, despite the omnipresence of these animals. Another interpretation could be that, as these dogs are everywhere, the mother had no way of preventing her child from touching the animal, and so she may have allowed this behaviour despite being aware of the potential seriousness of a bite, resigned to the fact that there was little she could do about it.

One animal healthcare participant told an illustrative story about how misconceptions around rabies can be fatal:

‘I remember one story. It was in the [*redacted*] area, a very remote place, we had to walk for 2 hours down to the village. One man’s niece had been bitten by a dog, and the uncle was so angry that he grabbed the dog and killed it but at that time the rabid dog bit him. Whenever villagers told him to get vaccinated, he just said ‘how could that dog get rabies? I am so powerful, I won’t get rabies.’ His wife was telling us this[…]the uncle died.’ [Livestock6].

Members of a FGD demonstrated perceptions that can exist around symptoms of rabies once a person is bitten:

‘P1: They start to act like dogs.

P2: I also heard that they give [*birth to*] babies like dogs.

P3: My father-in-law was bitten by a dog and did not get vaccinated due to which he started salivating, also he would start barking and digging.’ [Gulmi FGD].

##### Livestock and disease

Most participants implied that healthy-looking animals would not transmit disease: if they look healthy, they are healthy. Some participants were unwilling to believe that they could catch diseases from animals, particularly as they are often in close contact with livestock, pet dogs, and synanthropic animals such as rodents and bats and have never experienced any concomitant illness:

‘I don’t have any idea [*about animal-human transmission*]. When we rear animals, they look healthy and we also have not been affected[…]Rats have been around us since a long time, we also see them in the field but we have never fallen sick. Do they really transmit disease?’ [Gulmi3].

‘[*Cattle*] may transmit disease but I have reared them and they are healthy and I don’t think they would transmit disease.’ [Gulmi4].

One participant stated that they take as much care of their animals as they do their children:

‘Maybe, it [*animals*] can transmit diseases but I have had no knowledge or information about it. I take care of all my animals as my child but have no idea of the disease it may cause.’ [Pokhara3].

##### Sources of information

One of the disease awareness sub-themes focused on where participants acquired their information. Sources of information were usually informal (e.g., social media, discussions with friends or relatives) although some participants did mention more formal routes (e.g., government awareness campaigns, NGO drama programmes). Three of the informal settlement participants enthusiastically discussed a drama group who came to their settlement and staged a play focused on health promotion and water, sanitation and hygiene issues.

The COVID-19 pandemic had raised awareness of disease transmission:

‘People did provide information [*on COVID-19*] but no programmes were conducted here. We usually listen to such information like washing our hand for 20 seconds in the TV and radio.’ [Gulmi3].

‘After the rise in COVID the use of social media has increased. We all got the awareness information about COVID through Facebook.’ [Bhaktapur FGD].

**Fig. 2:**
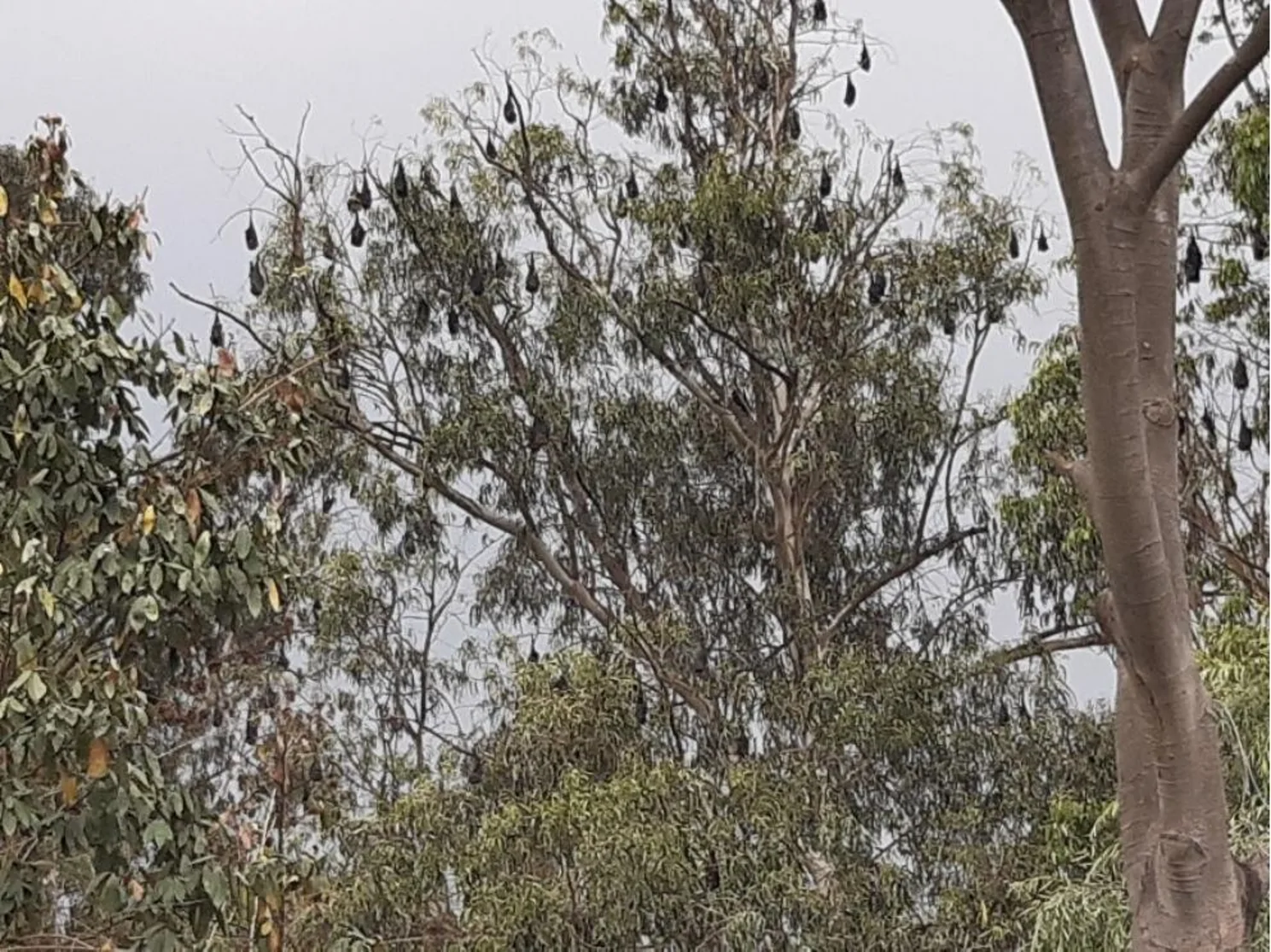
Fruit bats resting in trees in a public park (Bhaktapur4)

This raising of awareness was underlined by one of the photovoice images (Fig. 2), which was of a colony of fruit bats resting in trees in a widely used park in Bhaktapur, one of the major settlements in the Kathmandu valley. The participant who took this photograph stated that, before COVID-19, they would not have been aware of the potential for bats to affect humans but seeing these bats now in a public place, above their head, made them uncomfortable.

This was discussed further by a healthcare professional:

‘They wouldn’t be aware of many diseases, but they would be aware of like this bird flu, which makes the news, bigger news, that makes people fear, or bigger news that gets into the attention of the general public[…]Ministry of Health and Population, they also put out these notices through radio, newspaper and all those things about these diseases.’ [Health8].

One participant pointed out the importance of learning by experience, which was reflected in some participants’ experience with dog bites:

‘I have been hearing about animal-borne disease from TV, radio, friends and also have read in course book; however, I don’t give much more attention to such disease[…]I have been hearing such thing but have not such experience of animal disease. It’s said that you can learn best by experience so, due to lack of experience I don’t know more.’ [Pokhara10].

Underlining the point made above that awareness might be easier to spread if people or their animals are likely to be directly affected by a disease, one participant discussed an information campaign focused on keeping their livestock clean and healthy:

‘I have some idea as people come around to make us aware in every household, the municipality, to ask about the livestock and other information and also take names and make people aware of the disease they might cause as well as cleanliness and sanitation.’ [Chitwan5].

This was highlighted by a human health participant, who suggested that, during periods of elevated risk, people start listening:

‘At the lay person level, at the farming level, just the normal population of Nepal, the awareness about these zoonotic diseases is quite low. And periodically we do get to hear about avian flu, swine flu, H5N1, when that happens people suddenly become aware.’ [Health10].

However, as one healthcare participant pointed out, awareness is temporary and people forget once the initial outbreak or panic is over, so the question is how to ensure that this knowledge is retained:

‘Awareness can be quite temporary[…]. Each year we have an avian influenza outbreak in chickens, so at that time some people decide not to buy…even if it’s happening in some other province quite far away, sometimes people might have the tendency of not buying poultry items. But that is quite temporary. Even they can forget it after 1 week.’ [Health5].

#### Beliefs and behaviours

Under the second main theme ‘Beliefs and behaviours’, we included four sub-themes: traditional medicine use, bushmeat consumption, hygiene practices, and religious beliefs. Use of traditional medicine was widespread, although there was some discussion around whether this was an outdated practice, with some participants seeing this behaviour as pragmatic, as accessing traditional healers is often easier (and cheaper) than visiting health posts. These respected members of the community may have a central role to play in any activity designed to promote awareness of zoonotic (or any) disease, and aimed at addressing potentially risky behaviours (please see Discussion section). Consumption of bushmeat was seen as something that ‘others’ do, although some participants stated that bushmeat could be medicinal, and others dichotomised ‘clean’ and ‘dirty’ rodents. Hygiene practices were perceived as necessary to remove dirt, but this was not usually linked explicitly to illness. Religious beliefs were presented as a reason for not harming rodents through use of poison or traps.

##### Traditional medicine use

Many participants, male and female and of all ages, stated that, when ill, they would customarily use traditional medicine, and only consult an allopathic health professional if this did not work:

‘Most people try home remedies to cure certain diseases as it is our practice. Then if it does not work, we take them to the health post.’ [Mustang1].

‘We have visited the traditional healers in the past when my children were small because it was believed they can heal them. In case that did not work we would take them to the hospital.’ [Chitwan5].

Other participants discussed illness as being caused by spirits. If appeasing these spirits helps prevent or stop illness, allopathic medicine, which does not address this causation, will be ineffective:

‘People believe that there are certain spirits around the city and people sometimes get caught by them. The person then can suffer from stomach-ache or headache. And once you have made offering to these spirits it goes away.’ [Bhaktapur FGD].

‘P1: I have got bitten by dog. I went to a shaman [*dhami jhakri*]. I also took a ball of mud with me to show the bitten part. He then blew in it.

P2: Also, if snake bites you there is a different type of stone they use for the wound.

P3: These stones are still available but no one uses them as the shaman has already died[…]These stones suck all the poison.’ [Bhaktapur FGD].

Other participants suggested that visiting traditional healers was an outdated practice, dissociating themselves from older people and those in different, often rural, communities, and suggesting they are better educated and aware of modern medicine. Members of one FGD had an animated discussion about traditional healers and medicine, stating that, although older generations used to believe in traditional medicine, people now visit the doctor when ill and receive modern medical treatment. However, when probed, one participant explained that traditional medicines sometimes work better than allopathic treatment and so they still believe in their efficacy. Another participant discussed treatment of fractures, and that traditional medicines and mantras fix these better than allopathic treatments.

The urban-rural dichotomy was explicitly mentioned by one participant, with an implicit ‘othering’ of people in rural areas:

‘I have noticed that some people that do not get treated at the hospital get treated by the traditional healer. People do not believe these here but in the village a lot of people believe it. For example, some traditional healers take the poison from the wound of the snake bite with the help of mantras.’ [Pokhara FGD].

Echoing this othering, another participant made it clear to us during his interview that he identified as middle class and different from his neighbours; his family felt the need to ‘hide’ their visits to the healer from him, potentially a result of the perception of stigma attached to this practice:

‘I am against going to the traditional healers. But my family goes to them sometimes while hiding it from me. However, there are many people who believe in it.’ [Pokhara1].

Traditional medicines are also used to treat livestock in an attempt to avoid a time-consuming and potentially expensive (and futile) trip to the veterinarian:

‘If the goat had loose motion, we feed them hot oil in the winter. If the goat does not pee, we feed the leaf of the eggplant. If the homemade treatment does not work, we take them to the vet.’ [Gulmi2].

‘People ask us to take them to the vet but to get there it takes 3 to 4 days. Till then the buffaloes may already be healthy or have died due to the sickness. There are no proper services here. We give them garlic clove, mustard seed and other weeds. Hemp plants are also used.’ [Pokhara3].

One animal health professional discussed how (and why) communities would use traditional medicines in preference to allopathic treatments:

‘Sometimes they also give local treatment, locally available medicines, they don’t ask first[…]they just think like ‘ok, if this happens, last time this medication worked.’ It’s traditional, herbal medicine. And then they use that one. And sometimes even when we go there and we say ‘use this kind of medicine’ they say ‘no, this kind of herbal medicine works’[…]some farmers, because of their experience, they use herbal medication and if that does not work, it is severe, they then consult vets[…]people think there are lots of side-effects with modern medicines so they first choose the herbal medicines.’ [Livestock3].

Most communities have a resident traditional healer and people may deem it unnecessary to travel to a healthcare post that may not be open, or will be unaffordable, when they have accessible healthcare in their area. Traditional medicines are cheaper and easier to obtain than allopathic versions as they are often made from herbs that grow in the local area:

‘We want to know about the treatment as most of the people use herbs. Limited medicine is available so training on how to use such herbs could be given as not all the people can afford to buy the medicines.’ [Mustang FGD].

##### Bushmeat consumption

Participants were asked whether they knew of any people who had experience of eating bushmeat, and what they knew about these practices. Few participants admitted to having tried bushmeat themselves, although many of them knew of communities in Nepal (not their own – another example of othering) that did this. In this section, unless stated, participants were not of Tharu ethnicity:

‘I ate a snake but I never eat rats[…]We don’t eat rats, we don’t eat bats also. But you know in Tharu community they eat rats [*laughing*].’ [Kathmandu5].

‘In some of the community, from Terai they eat bat, many Chepang community eat. Tharu community eat rats. Like that in this community they didn’t eat bats and rats.’ [Mustang7].

One participant reluctantly admitted to eating rat meat:

‘I have eaten a rabbit. Okay, because rabbits are staying in the forest, forest rabbit. Before 10 years, I stay in another part of Chitwan, there was other Tharu people that they kill the rat. And then they make a meat and I taste it one time. Okay, I taste once.’ [Chitwan4].

A Tharu participant framed eating bushmeat as being an outdated practice, and something that people are embarrassed about:

‘No, we don’t [*eat rats*]. However, in earlier times people used to eat it as meat with wines and other. Still, very few of the population eat rats from field. Now they have stopped as people are educated and hesitate to eat them out of shyness as well.’ [Chitwan1].

Some participants judged bushmeat to be medicinal, or useful for disease prevention, although even in these cases participants were discussing the behaviour of ‘others’, and not their own or that of their community:

‘In the past we had heard the people did consume rat meats that they trap while harvesting rice in the paddy field. They believed that the mouse meat was medicinal.’ [Bhaktapur1].

‘People do eat rats and fox after finding it as it is believed to provide a lot of energy. People also traps rats in their house and eat it as well. People also believed that eating fox meat can cure or treat uric acid problems.’ [Gulmi2].

‘I have heard that people who suffer from piles eat mouse to treat it but I have not consumed it.’ [Pokhara3].

This issue was further clarified by a participant who is based in Kathmandu but spends time in a rural home. He explained the difference between ‘dirty’ and ‘clean’ rodents, focusing on the paddy harvest:

‘The Tharu people and still some people in my community love rats and eat rats. Because there are two kinds of rats, in my understanding. One rat that is available in the home and one rat that is available in the field[…]the one in the rice field, I think most of the people eat[…]the rats that are available in the cultivation field, they are a nicer one, cleaner, because they used to eat only rice that is produced in the field[…]they eat the clean rats but not the dirty ones.’ [Kathmandu5].

This was underscored by participants from other regions, who stated that field rats are ‘safe’ to eat (e.g., not likely to make the consumer ill):

‘As the rat in the field also eats grains so it is said that they are safe to eat.’ [Bhaktapur FGD].

This was reportedly even true of Tharu, who are perceived as traditional consumers of bushmeat by people outside this ethnic group:

‘These people [*Tharu*] do not eat the rats found inside the house but those rats which are found in the field by digging and trapping them. But no one eats rats in this area or this region.’ [Pokhara7].

Not eating rats was seen as a way of preventing disease by members of the Chitwan FGD, who stated that their ancestors used to eat rats and other bushmeat but stopped when they heard about ‘plague transmitted by such animals’.

The perceived dichotomy between ‘clean’ and ‘dirty’ rats was supported by an animal healthcare practitioner, who discussed rodent consumption at the end of the paddy harvest as a cultural event in the Nepali calendar:

‘Rat has many diseases. We have reports of leptospirosis in animals and in humans. So that might be a burden, but people they have a culture, they have a tradition that after they harvest the paddy, they keep on digging and digging to get the rats. They make the rat like a barbeque.’ [Livestock4].

##### Hygiene practices

Participants discussed various practices related to hygiene including food and water storage, and handwashing. Behaviours described tended to reflect beliefs around objects and animals being dirty rather than explicitly that lack of hygiene could spread disease. Many participants discussed the removal of rat faeces from, and thorough washing of, grain before cooking. One photovoice participant took a photograph of rat faeces mixed with grain in a container in their kitchen. When asked how they would deal with this situation, they said they would remove the faeces and then cook the grain:

‘The rats usually live under the cupboard and we see poop around there. We clean and throw it outside. We also clean the food and grains.’ [Bhaktapur2].

**Fig. 3:**
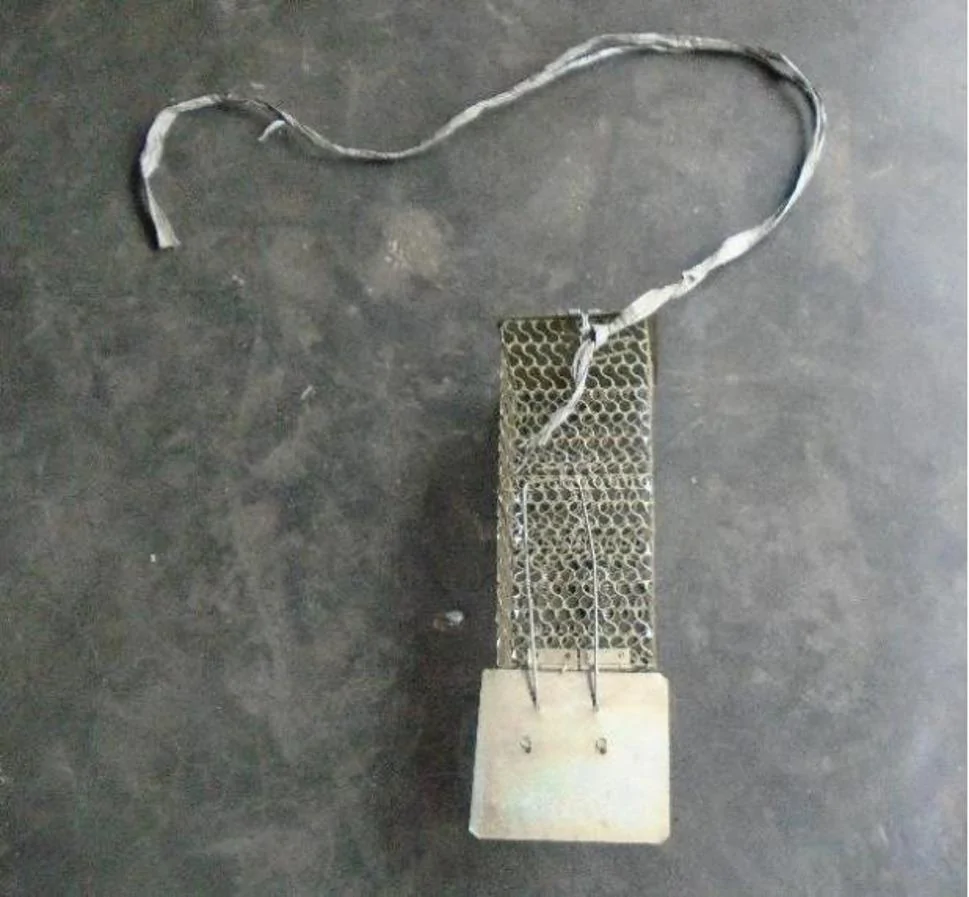
Rodent trap (Pokhara7)

Pokhara7 took a photograph of a rodent trap that he used in his kitchen (Fig. 3). This participant was aware that rats could spread disease:

‘I usually close all the rat holes inside the house and cover all the food that we consume. We usually try to control it as the rats can cause another disease. I usually don’t use poison and use mouse trap in which we use apple, peanut and pumpkin seed.’ [Pokhara7].

Participants boiled water and milk before consumption, even if they were unsure why they were asked to do so:

‘We do boil the milk first. First, we believed that after we drink unboiled milk we can get disease so we used to put salt in the milk.’ [Mustang FGD].

‘I started boiling the water as the doctors asked us to do so. I am not aware of the benefits it has.’ [Gulmi2].

Animal corpses were seen in and around water sources, and people were aware that this was a hygiene issue:

‘I have not seen dead rats around the house but what you have noticed is that those rats drown in the water pots and we find the dead bodies after we have consumed the water. Inside the big water tankers there is no filter[…]The rats drown there and decay and as we are unaware, we drink water from there.’ [Pokhara3].

**Fig. 4:**
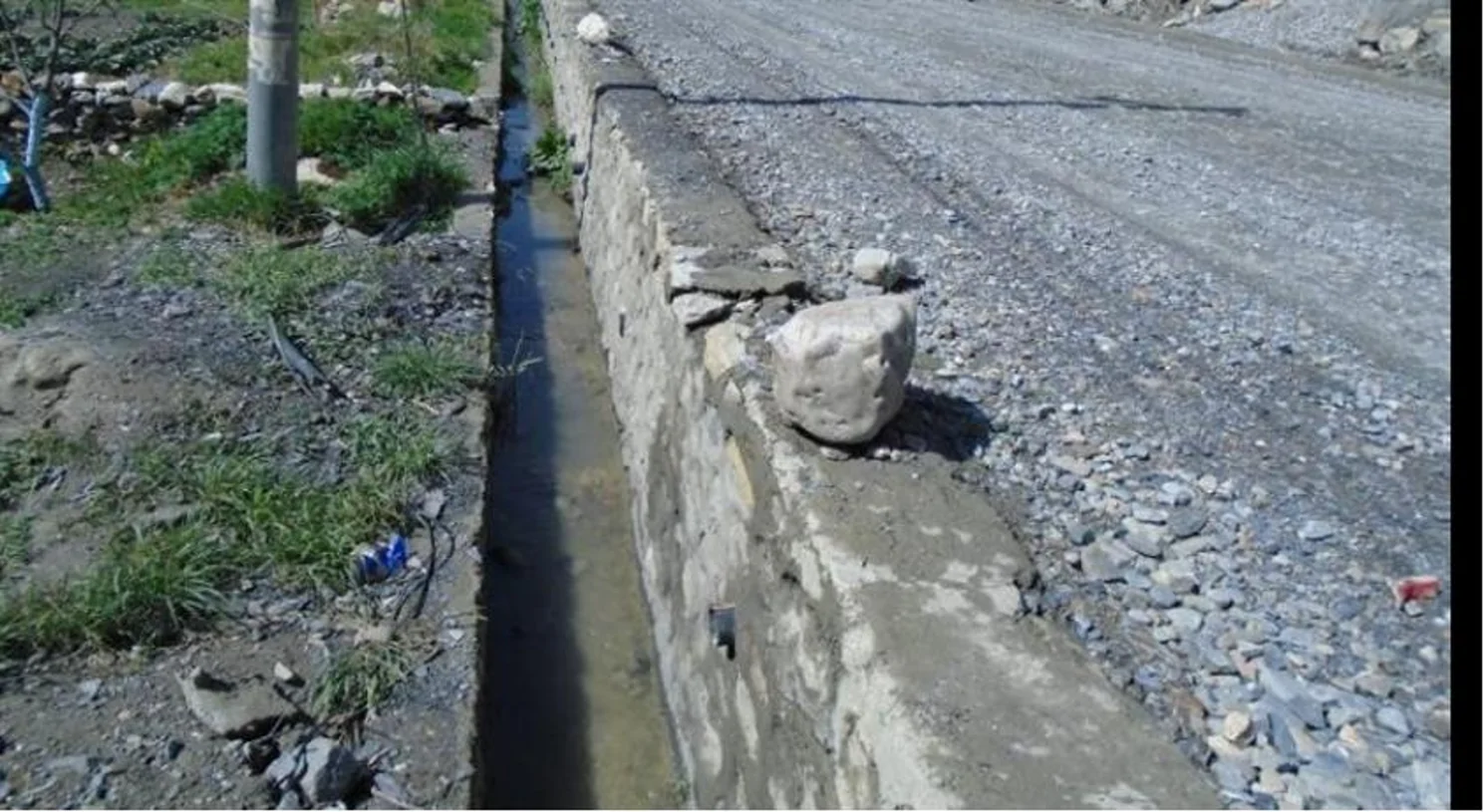
Polluted water course (Mustang1)

One participant in Mustang took a photograph of a water course running outside her home, along a main road and adjacent to her cultivated fields and animal shelters (Fig. 4). She stated that community members throw animal corpses into this water course, irrespective of how the animal died (e.g., poison, killed by feral dog, diseased domestic animal). She recognised the potential importance of this as a conduit for disease spread. This was supported by a comment from another participant in the same community, who clearly understood that the corpses then reached a larger water course:

‘I have seen dead bodies of the rats that we throw out in the water canal that connects to the river.’ [Mustang5].

Dead animals and disease were explicitly linked by a participant in Pokhara:

‘We find them [*dead rodents*] and throw it outside. Sometimes we also bury them because it’s said that it can also bring some kinds of diseases.’ [Pokhara6].

One participant said that they regularly ate food that had been contaminated by rats but that they had not experienced any ill effects:

‘[*Rats*] affects mostly all the crops. The mouse and the birds peck as well. We often eat rat-infected foods. I believe we don’t get ill by it. I don’t feel as if something’s happened to us till now.’ [Pokhara4].

However, others did suggest that consumption of food contaminated by rodents was likely to make them ill:

‘We wash it properly before consuming it. My mother used to tell me that if we eat rat poop our stomach would bloat so we need to clean the food properly.’ [Gulmi6].

Although washing hands after caring for livestock was perceived as important, domestic pets were perceived to be clean and therefore hygiene practices were unnecessary:

‘Yes, we do wash our hands [*after touching livestock*]. We also change our clothes if it gets dirty.’ [Chitwan6].

‘As he [*pet dog*] is domesticated, we do not wash our hand regularly.’ [Bhaktapur1].

Personal protective equipment (e.g., gloves, masks, boots) is not worn during rice transplanting in water-logged paddy fields, which can be a reservoir for leptospirosis spirochetes. Rice-planting is treated as a celebration by some community members. When asked about working in his parents’ paddy fields, one participant stated that planting the paddy was like a festival, everyone wearing their usual clothes and no protective gloves or boots:

‘We wear normal clothes […]I never heard of this [*leptospirosis*]. So usually in the rice field we used to do manually and it’s like a festival for us and we enjoy a lot in the mud, and we play like Holi [*water festival*]. It’s like a celebration.’ [Kathmandu5].

Other participants mentioned that boots are not used while transplanting paddy as it would make the process more difficult and therefore it is easier not to wear them:

‘No gloves but maybe boots when it is rainy but usually they work barefoot. It’s more I think comfortable for them without those things.’ [Bhaktapur4].

‘We work barefoot and with naked hands and feet.’ [Chitwan1].

Suggesting that it is partly a financial or availability issue, one participant stated that she would use protective clothes when looking after livestock if they were available:

‘I have not used [*gloves/boots*] yet. If such facilities are provided I am always ready to use them.’ [Gulmi5].

When protective clothing was used, e.g., when looking after animals, this was pragmatic, as a way of staying clean rather than about preventing disease spread:

‘I wear boots and slippers while growing corn, wheat, mustard and also when milking the cows to make sure we are clean but not to control disease.’ [Chitwan6].

##### Religious beliefs

Nepal is a deeply religious country, with the overwhelming majority of people professing to either Hinduism or Buddhism. One of the most popular gods of the Hindu pantheon, Ganesh, has a rodent (variously described as a mouse or rat) as a vehicle, while the first Buddhist precept is to refrain from killing any living being. These religious factors were mentioned by some participants as to why they would not harm rodents or other animals, and so would not use poisons or traps, but would instead live alongside them:

‘People who believe in Buddha do not allow to kill the rats as it is considered a sin.’ [Mustang FGD].

‘Some kinds of rats are not harmed as they as known as the vehicle of Lord Ganesh.’ [Pokhara11].

**Fig. 5:**
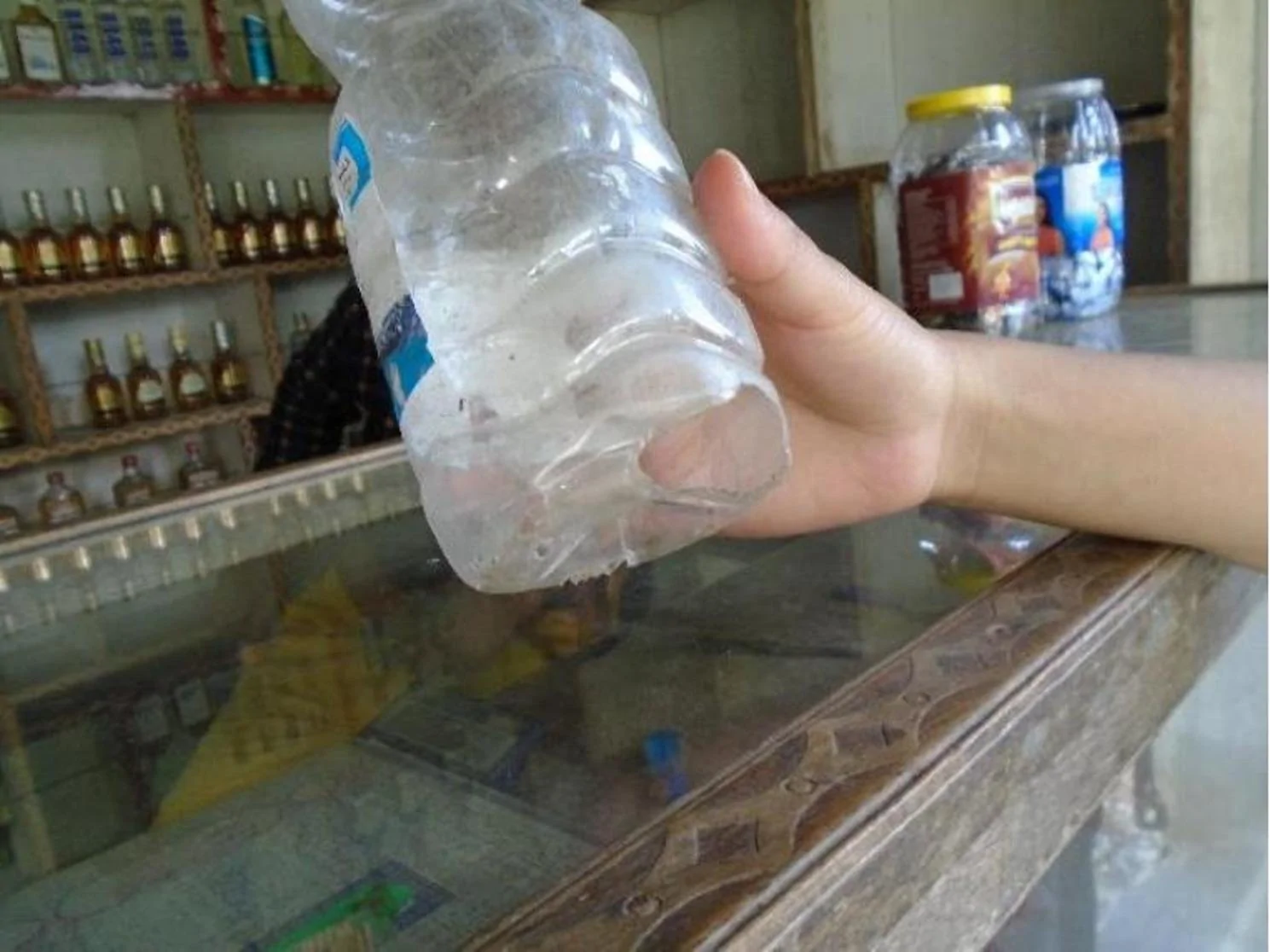
Rodent-damaged water bottle (Chitwan1)

One participant who runs a small roadside shop took a photograph of a water bottle from his stock that had been chewed and destroyed by a rodent (Fig. 5). Despite the negative effect on his income, he stated that he did not kill rodents due to his beliefs:

‘We haven’t applied any measures. We worship Lord Shiva and do not believe in killing rats.’ [Chitwan1].

Another participant stated that they did not use poison as they were worried that children would come into contact with it, but also had another poignant point to make, identifying with the pest animal:

‘We are scared to use poison as there are many children in our family. Also, my mother is against killing them as they have a family as well. They eat the grains and go but we cannot shoot them or capture them.’ [Pokhara3].

## Discussion

### Key findings

We present our main findings and the implications of these in Table 3, and discuss these further below.

**Table 3:** Main findings and implications

Findings	Implications
• Participants wanted to know more about zoonotic disease and were enthusiastic about taking photographs	• Community members should be involved in programming around zoonotic disease, taking an active, directing role. Photovoice was an effective means of involving participants in the research
• Participants usually denied that they knew about zoonotic disease, but further discussion suggested that they did understand some facets of these, particularly of rabies	• People may be more aware of behaviours designed to decrease risk of illness than they think (e.g., vaccinating dogs, removing rodent faeces from grain), although they may not recognise these behaviours as driven by this awareness
• Rodent-human conflict was mentioned as a serious issue, but some participants discussed religious reasons for not harming these animals	• Cultural and/or religious beliefs must be considered when designing interventions
• Many participants had consumed bushmeat but were often reluctant to discuss this issue	• Participants can be encouraged to discuss ‘taboo’ beliefs, through process of deflection; normative beliefs must be considered when designing interventions
• Traditional medicine/healers were frequently discussed with regard to healthcare	• Traditional healers can take a leading role in promoting awareness of zoonotic disease, and how to address ‘risky’ behaviours around these diseases
• Some participants appeared unwilling to believe that interacting with their livestock could potentially make them ill	• Smallholders should be reassured that zoonotic disease is not a reflection on their animal husbandry skills so they feel comfortable participating

In this study we examined perceptions of zoonotic disease in communities in Nepal, working with community members to reach a deeper understanding of this relatively neglected area of research. There was little difference in perceptions of and perspectives on zoonotic disease between male and female or urban and rural participants. This is perhaps unsurprising as this was a new subject for most participants, despite the fact that they are potentially at risk of zoonotic disease through exposure to synanthropic animals (e.g., rats and bats), consumption of livestock and bushmeat, hygiene practices, lack of awareness of transmission routes, and reliance on traditional medicine, compounded by a lack of accessible and affordable healthcare. Some behaviours were suggested as more likely to be performed by older people or in rural areas, but when younger or more urban participants were probed further they sometimes opened up and stated that in fact they had also, for instance, eaten bushmeat, or used traditional healing practices rather than visit an allopathic practitioner. This suggests a process of othering, where we differentiate ourselves from others who are situated as intrinsically different, possibly when participants felt uncomfortable about discussion of a behaviour that they judged to be out-dated or potentially harmful, and did not want to be associated with behaviours or practices [48]. This was clear in one interview, where the participant was keen to emphasise his membership in the middle class, and associated this with not using traditional healers, despite the fact that his family (and others in his community) did so.

The key findings of this study reflect many of the findings from our earlier scoping review, including the lack of official awareness campaigns focused on zoonotic disease (despite an appetite to know more discussed by participants, who may have a better understanding of mechanisms of preventing illness than they realise), the importance of involving communities in development of awareness campaigns, and the centrality of considering socio-cultural and normative beliefs when designing these, for example reliance on traditional healers, to try and ensure that they are more likely to be effective [15]. Inclusion of figures such as traditional healers or religious leaders (reflected in the importance of cultural drivers for not harming ‘pest’ animals) should be involved from the beginning of research or initiatives aimed at increasing awareness or changing behaviours. This illustrates the fact that socio-cultural practices must be considered when thinking about disease prevention, rather than simply assuming that awareness will be reflected in behaviour.

Our analysis generated two broad themes, awareness, and beliefs and behaviours, with relevant sub-themes subsumed under these that further developed these ideas. This dichotomy reflected findings from earlier research, which clearly identified these themes as relevant to our study, and in the same country [19, 27, 49, 50], and from our scoping review, which suggested that awareness does not always (or often) inform behaviours, or lead to a change in beliefs [15]. This apparent lack of coherence must be considered when planning future research, especially that designed to inform healthcare programmes. Themes should capture important facets of the data (in this case, drawn from four methods: interviews, focus groups, unstructured observations, and photovoice), and, as Green and Thorogood state, there is no ‘magic number’ of themes or sub-themes that should be identified. It is more important that the themes accurately reflect the material, which we consider is the case for our study [44].

Earlier research examining awareness and practices related to zoonotic disease among smallholder farmers in Nepal found that 40/89 (45%) of farmers were not aware of potential transmission from livestock to people. Any potential mitigatory practices (e.g., hygiene and vaccination) were not necessarily related to this awareness [19]. We found that, although most participants suggested they had little knowledge of zoonotic diseases and potential transmission, they were often able to discuss symptoms and prevention mechanisms, such as vaccinating pet dogs, avoiding contact with community dogs, and removing rodent faeces from grain. This suggests that participants may have been implicitly reluctant to demonstrate their knowledge (or, as they may have perceived it, lack of knowledge) in front of outsiders or perceived experts [48]. Despite having knowledge about rabies transmission and prevention, they may have felt intimidated or shy, or did not want to say the ‘wrong thing’, despite assurances that all opinions and views were interesting to us. Participants were able to discuss decisions they made that would protect their health (albeit sometimes indirectly), at the same time as claiming that they did not know that animals can transmit disease to them. All participants in the informal settlement – three of whom had low literacy – were aware of rabies, even if they were not always sure of the animals responsible for transmission. Two of them mentioned prophylactic vaccination of dogs as preventing spread of rabies (one had a pet dog). That rabies is better understood than other zoonotic disease may be a reflection of available funding for awareness campaigns, often run by the government in partnership with NGOs, who may also finance these. It could also be that participants are unable to avoid knowledge of rabies, when community dogs that may carry the disease are ever-present in their environment.

Rodent-human conflict (destruction of crops, clothing and property, concerns around infection and disease spread by rats) was mentioned frequently during the interviews, and most participant photographs featured either rat-mediated damage or attempts to mitigate the damage (e.g., home-made traps, covers for grain stores). Rodent consumption was discussed many times, although usually as an activity performed by ‘others’ rather than by the participant themselves or their community or ethnic group. The idea of ‘clean’ and ‘dirty’ rats, the former acceptable as food while the latter are not, possibly reflects an instinctive and unspoken avoidance of disease: not eating ‘dirty’ rats is adaptive and likely to benefit those people who behave in this way [51]. This is supported by findings from Viet Nam and Cambodia, where one study found that rodent consumers perceive rats as healthy, nutritious and disease-free [8]. Rodents that eat grain from the field, and not general waste and detritus from homes, are clean and therefore healthy and consumable. People are exposed to rat faeces and urine in their homes, and so associate ‘these’ rats with dirt (and, indirectly, with disease). Participants were (implicitly) aware that they should clean their grain because the rat faeces makes the food ‘dirty’ and that this is a bad thing, but they did not often explicitly discuss a link between dirt and illness or disease during interviews. Additionally, as demonstrated by some of the photographs and descriptions in this study, cultural or religious beliefs have a role in control of rodents, a major pest in Nepal, and must be taken into account in any attempt to mitigate damage from rats. People may prefer to live with the damage or potential illness caused by rats rather than kill them.

Participants sometimes evinced reluctance to discuss certain issues. For example, bushmeat was frequently mentioned, but usually as something that other communities did, or that was done in the past. This potential reluctance needs to be unpicked further. Adler and Adler, who have studied this phenomenon in depth, suggest that reluctance is a function of potential embarrassment at discussing topics perceived as sensitive [52]. This makes sense in terms of participants not wanting to talk about, or admit to, bushmeat consumption, or use of traditional medicine. The taboo around rodent consumption, reflected by participants’ reluctance to discuss it in relation to themselves, means that it may be challenging to address or even discuss, as the first step will be encouraging people to admit that it happens (and that they may also have participated). Bushmeat consumption is an important tradition for some ethnic and cultural groups in Nepal and this free and easily accessible source of protein is likely to be useful for a resource-poor community of subsistence farmers. As such it may be difficult to persuade people that eating rats or bats is not necessarily a healthy practice, especially as rodent consumption is associated with celebrations such as the paddy harvesting season. Kurpiers and colleagues, in discussing the effects of bushmeat consumption on emerging infectious diseases, suggest that disease spillover can occur through indirect contact (e.g., through exposure to faeces or urine) or consumption. Discussing rodents specifically, they suggest that Mpox virus, leptospirosis, salmonella, Lassa fever, and Mokola virus, have all been linked with consumption of rodent bushmeat [53–55]. Huong et al. discuss the relevance of the wildlife supply chain, bringing wild animals into restaurants for consumption, and state that this was a factor in the spread of COVID-19 within China and then to the wider world [8]. They found that positivity for coronavirus detection in field rats in Viet Nam increased along the supply chain: rats bought from traders were less likely to test positive than rats bought from large markets, which were less likely to test positive than those served in restaurants [8]. These studies support the case that eating bushmeat, or butchering wild animals, is likely to have deleterious effects on health, and at different stages from capture to consumption.

There was a distinct dichotomy between ‘us’ (me and our community) and ‘them’ (others who eat bushmeat, or ancestors who did so in the past). We observed much laughter and shyness around this issue, suggesting that some participants may have tried bushmeat but did not want to admit to it as it is judged taboo and perpetrated by ‘others’ who are in some way different. As rodents and bats are an excellent and readily available source of protein, another possibility is that by denying that they ate bushmeat, participants were inferring that they were rich enough to buy meat or to rear animals to eat. To place this in context, the communities described as eating rodents are judged poor and badly educated, often with low literacy, by some groups in Nepali society, and so admitting to a behaviour that these communities perform would be tantamount to placing the speaker within that group (or at least having something in common with that group). It must be kept in mind that, with the exception of one Tharu participant quoted above, most participants who discussed bushmeat practices were not of Tharu ethnicity, and this may have affected their perceptions of this ‘other’ grouping, especially as, as mentioned above, Tharu and other groups judged to belong to a low caste and believed to eat bushmeat (e.g., the Musahar community) are seen as ‘other’ and potentially stigmatised to an extent for this perceived activity. Interestingly, one of the participants in the informal settlement in Kathmandu was of Tharu ethnicity, a group widely believed to eat bushmeat. This participant stated that while Tharu in the west of Nepal will eat rodent meat, those in the east (and by definition, she herself) would usually not, although during festivals they may do so. It is also possible that participants did not want to discuss the eating of wild animals as hunting and killing these animals (e.g., wild boar or deer in protected wildlife reserves) is illegal in Nepal and so they may not have wanted to admit to behaviours that they knew could get them into trouble.

In terms of traditional medicine, a belief in spirits who control health and sickness, as evinced by participants in the study who discussed visiting a dhami jhakri (shaman) to cure illness, means that people might be fatalistic about experiencing illness. Suggesting that they have agency over whether their behaviours affect the likelihood of becoming ill may be difficult. This relationship between belief in spirits and health has been investigated in Nepal in the context of tuberculosis [50], and perinatal mortality and morbidity [49]. Marahatta and colleagues found that, when sick, people with tuberculosis visited traditional healers in preference to allopathic practitioners, and this was related to their belief that their disease was a result of karma and so little could be done to address their health issue [50]. Similarly, Paudel et al. suggest that perinatal deaths were attributed to karma, fate, destiny and the will of the gods, and as traditional healers are perceived as chosen by the gods, these are the people who must be consulted to prevent such deaths [49]. A study in Laos found that health-seeking behaviour by community members was influenced by their belief in the healing power of rituals, spirits and traditional healers, and that these have an important psychological and social significance [56]. These belief systems have implications for any method of raising awareness, especially as, in many contexts, attending a healthcare centre and being prescribed allopathic medicines will be more expensive than visiting a traditional healer and using ingredients that can be picked in the local area.

Some participants suggested that traditional healers were an anachronism, dissociating themselves from older people and those in different, often rural, communities, and suggesting they are better educated and aware of modern medicine. Members of one FGD had an animated discussion about traditional healers and medicine, stating that, although older generations used to believe in traditional medicine, people now visit the doctor when ill and receive modern medical treatment. However, when probed, one participant reluctantly explained that traditional medicines sometimes work better than allopathic treatment and so they still believe in their efficacy.

A study in Bangladesh found that 59% of people bitten by dogs visited traditional healers before going to hospital [57], and in India 64% of people who had a dog bite adopted ‘religious practices’ to prevent rabies, rather than attending an allopathic health professional [58]. The consistent mention of traditional medicine as the first option for treatment when people were sick suggests that these practitioners are generally prominent, trusted and respected, as well as being easily accessible. Working with these healers and other community leaders in a way that is sympathetic to cultural beliefs and existing practices may be an effective avenue to increasing awareness in communities [49]. This has been demonstrated in studies in Ethiopia, Ghana, Mexico, Bangladesh, Mozambique [59–63], and Nepal [64], where training of traditional healers on issues around transmission and prevention of HIV resulted in a significant improvement in healers’ knowledge of these issues, facilitated provision of culturally acceptable education to local communities, and reduced the stigma around HIV/AIDS [64].

### Implications

Our findings have some clear implications for policy and practice in Nepal. An understanding of community awareness, beliefs and behaviours, as told by communities themselves, is essential to work toward co-production of a contextually relevant intervention that will have resonance in the community [24]. As one of the human healthcare professionals stated, emphasising the importance of awareness, ‘Just because people are not aware of something, it does not mean that they are not getting it’ [Health5]. Many participants were aware of behaviours that might put them at risk of disease transmission, for example, dogs and rabies. We need to work with communities to ensure that potential routes to awareness reflect what people know, what they want to know, and what is feasible in their situation, to increase the likelihood of interventions and policies being effective for the people they are aimed at.

With the exception of one older participant, all participants stated that they wanted to know more about what actions they could take to avoid zoonotic disease. While we acknowledge that such responses may have been, at least to an extent, a result of participants wanting to be polite and respectful to the researchers, most participants appeared genuinely happy to be asked to take part, with many saying that they had never been asked for their opinions before. This was especially true of one participant who was asked to take photographs and became so enthusiastic that he kept bringing out more drinks so that we would stay longer, and he could take more images. This suggests that there is an appetite for awareness programmes, particularly when people can take an active, participatory role in them, and this willingness to participate could be fostered by organisations.

Photovoice was effective in getting participants involved in sharing their views. It was an ideal method to give participants an opportunity to not just talk, but to do something they felt was constructive to illustrate their thoughts. For the researchers, it was enriching to be able to visualise participant priorities, hearing in their own words and seeing through their eyes, as far as possible. People in the communities we visited were enthusiastic about taking photographs once we explained why we wanted them to do so. This was despite the fact that many of them had not used a camera before and were not sure what to do with it. Nobody refused to take photographs and nobody had to deliberate for long on what they wanted to show us. Most participants said that they appreciated the opportunity as they had never been asked to voice their opinion in this way. Photovoice has been used as a method of empowering participants to speak [65], and encouraging discussion around sensitive subjects [36]. This was reflected in our study. One participant from the informal settlement, a marginalised group in Nepal, was enthusiastic about taking photographs and produced one of the most informative descriptions of the whole study. Including underserved populations who are rarely, if ever, given the opportunity to discuss their opinion, was a rich source of information and will likely suggest ways of being more inclusive when designing initiatives [66].

Awareness was present to some degree despite a lack of formal educative programmes or campaigns run by local or federal government. Three participants living in the informal settlement talked enthusiastically about a drama session run by an NGO, which had obviously resonated with them, suggesting that this may be a good way to involve community members in programmes around hygiene or related issues, like zoonotic disease transmission. Participants enjoyed these sessions, particularly appreciating the fact that an organisation had spent time and resources coming to their settlement to run the awareness campaign. Events run inside the settlement, and therefore not requiring any financial outlay from the participants, who may not have the financial resources to attend clinics, may be especially useful. Visual theatre, rather than written notices, worked well in this situation as most settlement residents have low literacy: of the four participants, three had at most two years of schooling and stated that they were unable to read or write. This suggests that an effective awareness campaign might benefit from some interaction or role-playing, so participants can imagine what a dog bite feels like, and what kind of action they are able to take in this event. Drama and theatre have been demonstrated to promote community awareness, engagement, and empowerment in communities that are underserved and likely to have low literacy [67], as in the informal settlement. Another potential avenue for raising awareness has been demonstrated by the PREDICT project, supported by the United States Agency for International Development and focused on detecting, preventing and controlling infectious disease risk in people and animals globally [68]. This project includes social scientists, who are tasked with identifying behaviours in communities at the human-animal-environment interface, which are judged high risk for virus emergence. Working with local leaders from various countries, the team produced a picture book with simple text, ‘Living safely with bats’, that illustrates the importance of bats to local ecosystems, their role as a potential disease vector, and how community members can live safely with these animals [69]. During community meetings, a local leader presents the book and discusses the images with the audience, creating awareness in a familiar space in which people are encouraged to ask questions and share their opinions [70].

Unlike the perceived difference between ‘clean’ and ‘dirty’ rats, there is no similar split between domestic pets and livestock. This may be related to the close relationship between people and their animals described above. Some participants appeared to take the issue of zoonotic disease personally – they take good care of their animals and so they would not be responsible for causing illness. These participants took obvious pride in rearing their animals and looking after them, and may have felt that we were questioning their animal husbandry skills, although we worked hard to make it clear that we were in no way judging any of their practices. For many of the participants in rural areas, their livestock will be their livelihood and a huge investment. They may not want to consider the ramifications on their income of their animals becoming ill and dying, or their reputation in the village if their animals are believed to be causing illness, as well as having an emotional bond with their animals. Research suggests that members of poorer communities who raise livestock spend more money on the health of their animals than on their own health, particularly on therapy rather than prevention [71], so there is definitely space for more research to pinpoint what would encourage smallholders to learn about zoonotic disease, without perceiving this as a reflection on their animal husbandry skills.

As described above, many participants were reticent to discuss bushmeat consumption when it referred to their own practices rather than those of others. The taboo nature of bushmeat consumption may complicate interventions to address the practice, as identifying and then working with consumers will not be straightforward if they do not feel comfortable admitting (or even acknowledging) the practice. When we realised that people were not comfortable with discussing this issue, we used deflection, which involves deflecting attention from the participant, and instead discussing a topic in a more general way [52]. Instead of asking directly whether they themselves ate or had ever eaten bushmeat, we asked whether they had ever heard of any person or community in Nepal who practised this. This approach was effective, as participants often relaxed and then opened up about their personal experiences, with less evident discomfort. Ensuring that community members feel comfortable discussing these types of issues is the first step to addressing potentially significant consequences of these behaviours through appropriate mitigation strategies.

The unwillingness shown by some participants to think through the potential effects of zoonotic disease may be linked to fear, which was mentioned by one healthcare professional as a framing that might be an effective driver of awareness. However, fear can be counterproductive, and, instead of promoting healthy behaviours, may work against them. This was seen in the 2014–2016 Ebola virus outbreak in west Africa, where some fear-related behaviours increased risk of infection (e.g., hiding ill relatives or removing them from in-patient care, increased stigmatisation), when people were not clearly informed of the likely outcome of these behaviours (e.g., more people becoming infected) [72].

### Study strengths and limitations

Limitations of the study include that participants may not have felt able to discuss certain behaviours or practices that they perceived as sensitive or anachronistic (e.g., bushmeat consumption, use of traditional medicine), especially with an obvious outsider. However, as described above, we worked hard to deflect attention away from the fact that we were discussing this topic, and this did appear to work as people opened up to us. Our study sample was necessarily focused on certain areas, and people in other communities may hold different views. Although this limits the generalisability of our findings to other rural, peri-urban and urban communities across Nepal, we did find that opinions and practices were coherent across the different sites. Although our findings may not be applicable to communities in other contexts (both within Nepal and in other countries), we believe that they may be useful as a basis for informing further research that seeks to examine related topics or ideas, and readers must judge the relevance of our findings for the context in which they work. Interviewing was new to most participants, who had never been asked their opinions on this (or any other) topic before, so they may have felt uncomfortable with the process. While interviewing we worked hard to build a rapport with participants, explaining what we were doing and why, and discussing how their experiences and thoughts were important for the study. Community interviews almost all involved simultaneous translation and some meaning may have been lost during this process. To mitigate this, all recordings were transcribed verbatim and so transcripts included both the original Nepali vocabulary (translated into English) and the simultaneously translated English. Shared demographics may act as a link between interviewer and participant [52] and the first author was obviously a different ethnicity to all participants; although, as discussed in the Reflexivity section above, during the planning, interviewing, analysis and writing stages she attempted to keep her positionality, assumptions, preconceptions, values and motivations for doing the research in mind, there is a limit to how much difference this might practically make. Our methods were appropriate and robust and included different ways of gaining pertinent information, including interviews, group discussions and photovoice, a process which participants appeared to enjoy. With these caveats, we believe this study makes a meaningful and useful contribution to the limited body of evidence on awareness and behaviours around zoonotic disease in selected Nepali communities.

## Conclusion

This study demonstrated that a clear concept of why people behave as they do, through talking directly to the people involved and comprehending contextual factors, is necessary to effectively working to address threats that exist in these contexts and environments. Although we included participants from six communities, we recognise that this necessary focus may limit the generalisability of the findings, as people from different castes and ethnicities may experience the world differently. However, we identified common beliefs and experiences in these settings, the implications of which may be broadly relevant to other communities. Our findings that bushmeat consumption and, in particular, use of traditional medicine are relatively common practices must be placed into context. If people eat bushmeat because it is an easily accessible form of protein that has traditionally been used to cure illness, and if traditional medicines are used because healers are trusted members of the community, and, again, more easily accessible than health posts, then these drivers must be taken into consideration in any attempt to understand a community’s perspective on illness and what can be done to address these threats. Understanding how people perceive potential disease threats, and how their behaviours may influence these, is key to beginning to address these threats. Working with communities and understanding explicit and implicit contextual factors around behaviours is essential to unpicking the complexities that might inadvertently encourage the spread of diseases, especially in communities that lack resources to mitigate these threats. Specific implications for policymakers are recognising the need to ensure that the healthcare system is inclusive of the different groups which access its services, and that different beliefs are taken into account (e.g., incorporation of traditional medicine practitioners into more formal healthcare programming). Future research could examine gender-, age- or ethnicity-specific responses to threats from zoonotic disease, which may affect effective policy-making.

## Supplementary Information


Supplementary Material 1.


## Data Availability

All relevant data generated or analysed during this study are included in this published paper.
